# Novel Hypertrophic Cardiomyopathy Diagnosis Index Using Deep Features and Local Directional Pattern Techniques

**DOI:** 10.3390/jimaging8040102

**Published:** 2022-04-06

**Authors:** Anjan Gudigar, U. Raghavendra, Jyothi Samanth, Chinmay Dharmik, Mokshagna Rohit Gangavarapu, Krishnananda Nayak, Edward J. Ciaccio, Ru-San Tan, Filippo Molinari, U. Rajendra Acharya

**Affiliations:** 1 Department of Instrumentation and Control Engineering, Manipal Institute of Technology, Manipal Academy of Higher Education, Manipal 576104, India; anjan.gudigar@manipal.edu (A.G.); chinmaydharmik@gmail.com (C.D.); rohit4gm@gmail.com (M.R.G.); 2 Department of Cardiovascular Technology, Manipal College of Health Professions, Manipal Academy of Higher Education, Manipal 576104, India; samanth.jyothi@manipal.edu (J.S.); krishnananda.n@manipal.edu (K.N.); 3 Department of Medicine, Division of Cardiology, Columbia University Medical Center, New York, NY 10032, USA; edwardciaccio@gmail.com; 4 Department of Cardiology, National Heart Centre Singapore, Singapore 169609, Singapore; tanrsnhc@gmail.com; 5 Duke-NUS Medical School, Singapore 169857, Singapore; 6 Department of Electronics and Telecommunications, Politecnico di Torino, 10129 Torino, Italy; filippo.molinari@polito.it; 7 School of Engineering, Ngee Ann Polytechnic, Clementi, Singapore 599489, Singapore; aru@np.edu.sg; 8 Department of Biomedical Informatics and Medical Engineering, Asia University, Taichung 41354, Taiwan; 9 International Research Organization for Advanced Science and Technology (IROAST), Kumamoto University, Kumamoto 8608555, Japan; 10 Department of Biomedical Engineering, School of Science and Technology, SUSS University, Singapore 599494, Singapore

**Keywords:** computer-aided diagnosis tool, deep features, hypertrophic cardiomyopathy, integrated index, ResNet-50

## Abstract

Hypertrophic cardiomyopathy (HCM) is a genetic disorder that exhibits a wide spectrum of clinical presentations, including sudden death. Early diagnosis and intervention may avert the latter. Left ventricular hypertrophy on heart imaging is an important diagnostic criterion for HCM, and the most common imaging modality is heart ultrasound (US). The US is operator-dependent, and its interpretation is subject to human error and variability. We proposed an automated computer-aided diagnostic tool to discriminate HCM from healthy subjects on US images. We used a local directional pattern and the ResNet-50 pretrained network to classify heart US images acquired from 62 known HCM patients and 101 healthy subjects. Deep features were ranked using Student’s *t*-test, and the most significant feature (*SigFea*) was identified. An integrated index derived from the simulation was defined as $100\cdot log_{10}\left(SigFea/\sqrt{2}\right)\;$ in each subject, and a diagnostic threshold value was empirically calculated as the mean of the minimum and maximum integrated indices among HCM and healthy subjects, respectively. An integrated index above a threshold of 0.5 separated HCM from healthy subjects with 100% accuracy in our test dataset.

## 1. Introduction

**Figure 1:**
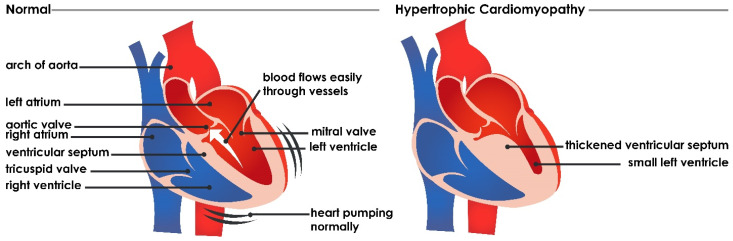
Coronal sections of the heart depicting morphological differences in normal versus hypertrophic cardiomyopathy.

Hypertrophic cardiomyopathy (HCM) is an autosomal dominant genetic disorder caused by mutation of one of several genes coding for various proteins of the cardiac sarcomere. Morphologically, HCM is characterized by a hypertrophied, nondilated left ventricle with or without right ventricular involvement in the absence of another cardiac or systemic disease [1,2]. The prevalence of HCM is approximately 1 in 500 persons in the general population [3]. Altered sarcomeric proteoforms have been identified in surgical samples of HCM patients [4], and the hypertrophied wall is composed of disarrayed myocardial fibers with interstitial fibrosis, which results in reduced ventricular compliance [5] (see Figure 1). HCM may exhibit clinical vacillation from asymptomatic to sudden cardiac death in young adults [6,7,8].

On the cardiac ultrasound (US), the classical form of HCM is typified by asymmetrical septal hypertrophy, which is defined as an interventricular septal (IVS) thickness of at least 15 mm and increased ratio of the IVS to left ventricular posterior wall thicknesses > 1.3 in the absence of any valve or systemic disease [9]. In other forms, hypertrophy may involve different myocardial segments. HCM can be classified into reverse curvature septum, sigmoid septum, neutral, apical HCM, and midventricular hypertrophy subtypes [10]. Pathophysiologically, this results in both systolic and diastolic left ventricular dysfunction [11]. This study aimed to develop an automated detection method to discriminate HCM versus healthy controls on the cardiac US.

Computer-aided diagnostic (CAD) tools are increasingly being used to reduce the time-cost of diagnosis. In [12], a CAD tool for diagnosing congestive heart failure (CHF) on electrocardiogram (ECG) signals was described. Other researchers used CAD tools to analyze US images. The use of CAD tools to categorize infarcted myocardium versus normal on heart US images [13] and artificial intelligence to analyze cardiovascular US images in general [14] have been reviewed. In [15], the authors extracted textural features and employed particle swarm optimization, attaining a maximum accuracy of 99.33% for diagnosing CHF. The same group used the double-density dual-tree discrete wavelet transform (DD-DTDWT) to identify coronary artery disease, achieving an accuracy of 96.05% [16]. They extended their work to screening pulmonary hypertension using entropy features and attained a classification accuracy of 92% [17]. Notably, the same group has developed a CAD tool to recognize the four-chamber heart US images of the fetuses of pregnant women with pregestational diabetes mellitus or gestational diabetes mellitus using the local preserving class separation technique [18].

Very few works have been published on the automated characterization of HCM or left ventricular hypertrophy using US images [19,20,21,22]. In [19], dilated cardiomyopathy and HCM were diagnosed from heart US parasternal short-axis views. The left ventricle was segmented using fuzzy c-means clustering, and features were extracted using principal component analysis and discrete cosine transform, which were then fed to various classifiers. An overall accuracy of 92.04% was achieved for classifying normal versus abnormal hearts using principal component analysis (PCA) features with the backpropagation neural network (BPNN) classifier. In [20], Darwinian particle swarm optimization (DPSO) and fuzzy c-means (FCM) clustering were used for segmenting the left ventricle on the parasternal short-axis view. For the extracted gray level co-occurrence matrix (GLCM) and discrete cosine transform (DCT) features, 90% accuracy was achieved using a support vector machine (SVM) classifier. In [21], a multilayer convolutional neural network (CNN) model was trained to detect HCM using the apical four-chamber view, which achieved high discriminant utility with a C statistic value of 0.93. In [22], texture-based analysis was utilized to characterize HCM by first-order statistics, and the GLCM, along with the features, were fed to an SVM classifier.

The current work developed CAD tools for assessing HCM using four-chamber heart US images. The contributions of the paper are as follows:

- Established databanks of four-chamber US images of normal and HCM subjects;
- Created deep features by combining local texture featured images with deep neural networks; and
- Generated an integrated index to categorize normal versus HCM using a distinctive number.

The remainder of the paper is organized as follows: Section 2 describes the materials used and the US image acquisition. Our analysis methodology is outlined in Section 3. Experimental results and discussion of the results are presented in Section 4 and Section 5, respectively. Finally, the concluding remarks of the paper are given in Section 6.

## 2. Materials

**Figure 2:**
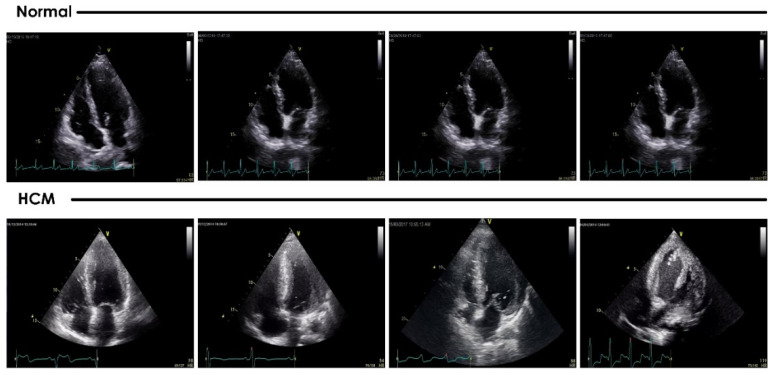
Example apical 4-chamber US images of normal versus hypertrophic cardiomyopathy participants.

A total of 62 (mean age 50.7 ± 14.3 years) patients diagnosed with HCM who visited the cardiology outpatient department at a single center were prospectively recruited, and 101 age-matched healthy individuals (mean age 52.4 ± 15.5 years) attending the same center for routine health checks were recruited as controls. The institutional ethics committee approved the study at Kasturba Hospital Manipal (IEC NO.: 48/2020), and informed consent was obtained from the participants. HCM was diagnosed if the echocardiographic examinations showed a nondilated, hypertrophic left ventricle (LV) without any known cause; i.e., long-term hypertension or another cardiac/systemic disease, and the ratio of the thickness of IVS and posterior wall thickness (PW) was >1.3 with or without left ventricular outflow tract obstruction (LVOTO), or patients diagnosed with apical HCM. Among HCM patients, 42 (67.74%) presented with symptoms such as chest pain, dyspnea on exertion, and syncope, and 20 (32.25%) were incidentally diagnosed with HCM. Subjects with hypertension, renal failure requiring medical intervention, left ventricular ejection fraction < 55%, known ischemic heart disease, congenital heart disease, and valvular heart disease of more than mild severity were excluded from the study. All participants underwent heart US examination on a Vivid S60 system (GE Healthcare) with a 3Sc-RS phased array transducer probe and a frequency range of 1.3 to 4.5 MHz. Standard parasternal short-axis view at the mid-left ventricular (papillary muscle) level and apical 2- and 4-chamber views were acquired and archived digitally. In each participant, one static image at one time frame corresponding to the R wave on ECG from the cine 4-chamber view was selected for analysis using the CAD. In total, 62 and 101 images of HCM and normal subjects, respectively, were analyzed. Examples of typical images used are provided in Figure 2.

## 3. Methodology

**Figure 3:**
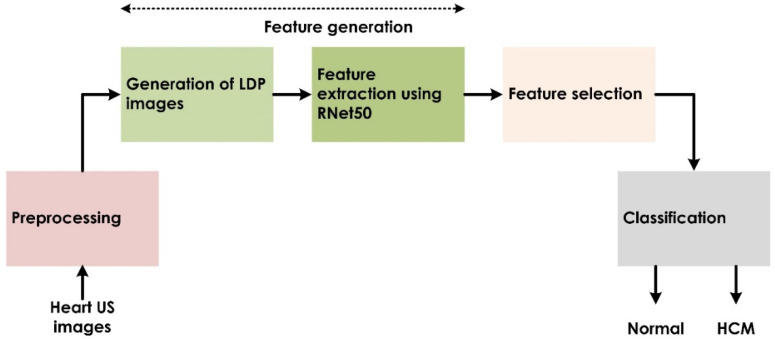
Schema of the deep-features-based proposed architecture.

Deep neural networks have achieved excellent performance for pattern recognition [23,24,25,26]. Such a model is typically trained on a large dataset in one domain, and the knowledge gained is then transferred to another domain comprising a smaller dataset [27]. In the current study, we exploited local descriptors such as the local directional pattern (*LDP*) [28] and a pretrained ResNet-50 (RNet50) [29] network to generate deep features. Figure 3 shows the various stages of the proposed system, which include feature generation, feature selection, and classification. A detailed description of each stage is provided in subsequent sections.

### 3.1. Preprocessing

**Figure 4:**
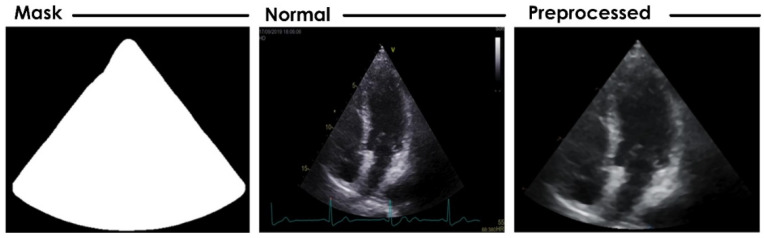
Preprocessed image derived by creating a mask.

Unwanted information such as labels, signals, etc., was first removed from the apical 4-chamber heart US image. A mask was then generated to extract the region of interest to increase system efficacy, and a median filter of size 5 × 5 was applied to it to reduce the noise level. The filtered image was then resized using the bicubic interpolation technique for further processing [30]. Figure 4 shows the created mask and preprocessed image.

### 3.2. Feature Generation

This stage generated the features used to characterize the 4-chamber heart US image.

#### 3.2.1. Local Directional Pattern (*LDP*)

The *LDP* is a descriptor that uses Kirsch compass kernels to extract the directional component [28], and is an improvement on the traditional local binary pattern. For a given central pixel $i_{c}$ of an image, with 3 × 3 neighborhood pixels of intensity values $i_{n}\;,\;n=0,1\ldots ,7$, Kirsch edge detectors of 3 × 3 with eight possible orientations centered at $\left(x_{c},\;y_{c}\right)$ were considered to obtain responses $M_{n},\;n=0,1,\ldots ,\;7$ corresponding to pixel value $i_{n}.$ Based on the $k^{th}$ Kirsch activation (i.e., $M_{k}$), all neighboring pixels with higher Kirsch responses were set to 1, while the rest were set to a null value, as we were only interested in generating the *LDP* pattern with the most evident directions. Then, the *LDP* value of $\left(x_{c},\;y_{c}\right)$ with various directional responses was given by:

(1) $$LDP=\sum_{n=0}^{7}b\left(M_{n}-M_{k}\right)\cdot 2^{n}$$

(2) $$b\left(x\right)=\{\begin{array}{c} 1;\;x\geq 0\\ \;0;\;otherwise \end{array}$$

**Figure 5:**
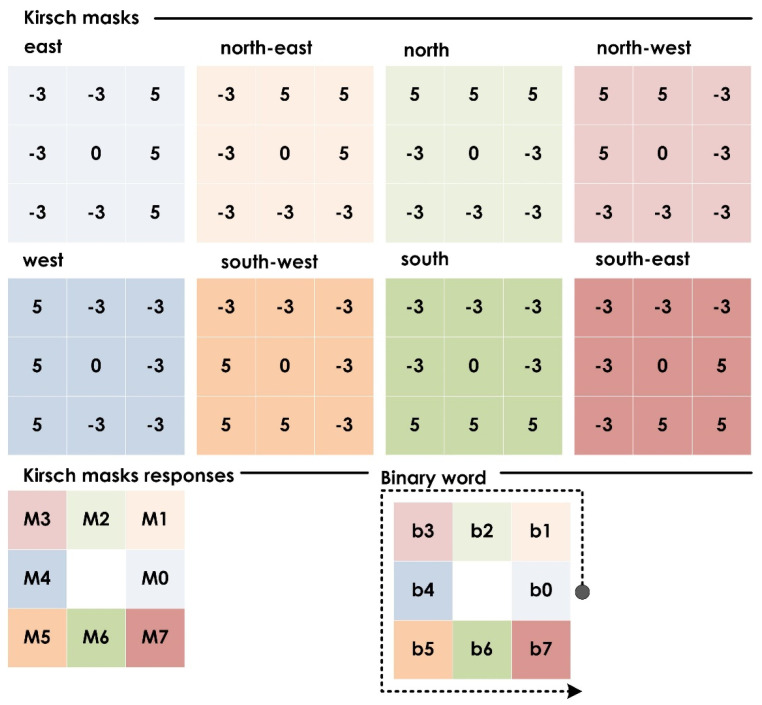
Computation of local directional pattern. Kirsch mask responses (M0, M1, M2, … M7) are obtained for a central pixel within the image with eight Kirsch masks that are rotated through the eight compass directions (east, north-east, north, … south-east). The neighboring pixel with the maximum Kirsch mask output determined the local direction. To form the local directional pattern, all eight neighboring pixels are sorted by the output (M0, M1, M2, … M7), and “one” and “zero” assigned to the highest and lowest four pixels, respectively (b0, b1, b2, … b7). The process is repeated throughout the image.

The complete process to compute the *LDP* is illustrated in Figure 5.

**Figure 6:**
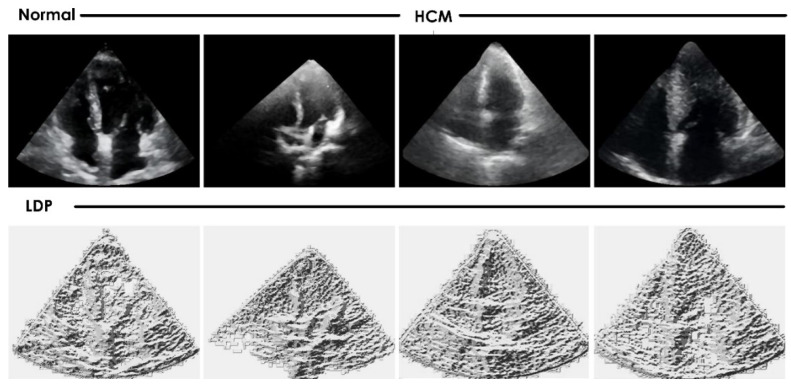
Preprocessed 4-chamber heart images and the corresponding *LDP* images.

The generated *LDP* patterns were more stable in the presence of noise and changes in image brightness. Figure 6 shows the preprocessed 4-chamber heart US images and the corresponding *LDP* images.

#### 3.2.2. Deep-Learning Model

RNet50 is a deep-learning neural network for image classification that has been pre-trained using a subset of images from the ImageNet database [31]. The network is based on residual learning, and comprises 50 layers. Deep networks facilitate the extraction of significant features for efficient classification [32]. RNet50 is a deeper network than other CNN architectures, yet possesses fewer parameters [33]. The stacking of convolutional layers usually demonstrates good performance initially, but later declines due to gradient vanishing [33,34]. RNet50 circumvents the degradation issue by incorporating a deep residual learning framework with identity mapping [33,34,35,36]. The latter allows the CNN model to bypass the present weight layer if not required; therefore, inputs from the present convolutional layer can be copied to the next one without any alteration. The residual block is fundamental to the RNet framework. RNet50 contains 16 such residual blocks, on which it capitalizes to accelerate the network’s training, reduce training errors, and preserve the accuracy [37]. The residual block for the considered stack of three layers was defined as:

(3) $$y=F\left(x,\left\{W_{i}\right\}\right)+x\;$$

where *F* is the residual function that needed to be learned, *x* is the input vector, and *y* is the output vector that could be obtained by performing element-wise addition and skip connection.

**Figure 7:**
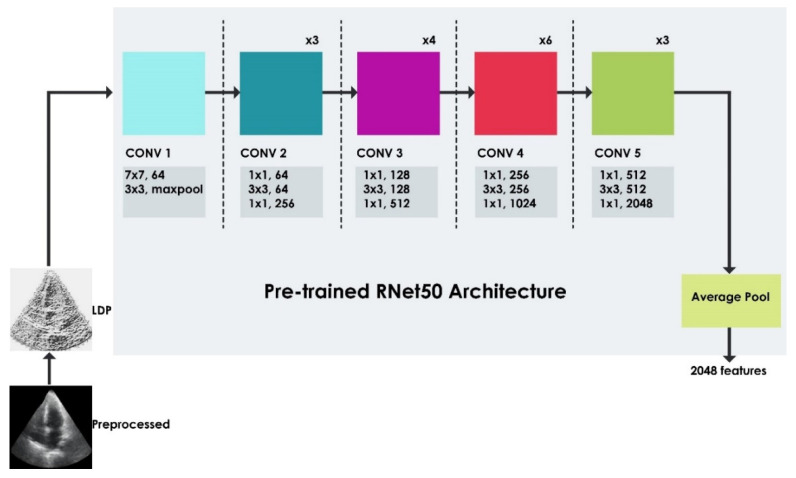
Feature generation blocks of the proposed method.

The bottleneck residual block for RNet50 consisted of three convolutional layers: the initial and final 1 × 1 layers reduced and restored the dimensions, respectively; while the middle 3 × 3 layer dealt with the reduced dimensions [29,38]. This bottleneck architecture greatly reduced the computational complexity and the number of parameters. The inputs to RNet50 were RGB images with dimensions of 224 × 224, and the output dimensions were reduced to 112 × 112, 56 × 56, 28 × 28, 14 × 14, 7 × 7, and 1 × 1 after passing in turn through the five categories of convolutional and average pool layers [29,39]. These RGB images were obtained by triplicating the gray level channel. In general, deep layers learn global features while shallow layers are utilized to capture features such as corners, edges, curves, etc. (i.e., local features). The knowledge of learned features from a general image dataset can be transferred to other domains, which helps save training effort and time [27,36,40,41]. The current study used the pretrained RNet50 with its weights unchanged to extract the local features from heart US images to train the final layers. Here, transfer learning was a nonlinear function that used the source task and domain knowledge to learn the target task in the target domain [23]. The complete structure of the feature extraction stage is illustrated in Figure 7. Note that the *LDP* (not the raw) image of the heart US image was input to the pretrained RNet50 to obtain a feature size of 2048. The RNet50 architecture had been pretrained in the ImageNet database [31] and modified to classify the images into two classes: normal and HCM. The weights were updated using the stochastic gradient descent with the momentum algorithm on a GTX 1070 GPU, with an initial learning rate of 0.0001 and a learn rate drop factor of 0.01. This was done for a mini-batch size of 32, and the sequence was shuffled every epoch and validated at every 10th epoch.

### 3.3. Feature Selection

A Student’s *t*-test was used for feature selection. This test measures the statistical difference between two sets by computing the ratio of the difference of the means between and the variability of the two classes [42]. The null hypothesis is that the means of two groups are equal, and the hypothesis can be rejected based on the computed *t*-value:

(4) t=μx−μy(σx2Sx)+(σy2Sy) 

where $\mu_{x}$ and $\mu_{y}$ and $\sigma_{x}$ and $\sigma_{y}$ are the means and standard deviations of the two groups, respectively, and $S_{x}$ and $S_{y}$ denote the total numbers of samples for the two groups [43]. The *t*-values and the corresponding *p*-values were calculated for the features of both classes. The *t*-value estimated the difference between the two groups, and a significant *p*-value implied that the difference had not occurred by chance. Features were ranked by selecting for higher *t*-values with lower *p*-values [44].

### 3.4. Classification

In this stage, the classes were predicted using the supervised and unsupervised methodology. Most medical applications use supervised classification techniques, such as probabilistic neural networks [45,46], SVM [47,48], *k*-nearest neighbor [49], etc., to predict the class label of target data. However, the parameter settings required by some of these classifiers may result in overfitting. To overcome this, many researchers have employed indexing to reflect the inference of generated features, whereby a distinct number is formulated to distinguish abnormal from normal images [50,51,52,53,54,55,56,57,58,59]. Accordingly, we formulated an integrated index for HCM (*IIHCM*) based on the significant features. The equations were empirically derived from simulation, and are given as:

(5) $$IIHCM=log_{10}\left(SigFea/\sqrt{2}\right)\times 100$$

and

(6) $$Th=\frac{\left\{\min\left(IIHCM_{hcm}\right)+\max\left(IIHCM_{normal}\right)\right\}}{2}$$

where SigFea is the most significant feature selected based on its highest *t*-value.

Dividing the feature by $\sqrt{2}$ would clutter the data with the same class, but the $log_{10}$ term would separate the classes to a great extent. The threshold value (*Th*) was calculated as the mean of the minimum and maximum integrated indices among HCM and healthy subjects, respectively, to obtain the definite boundary between the HCM and normal heart images; i.e., attaining discrimination between normal and HCM classes using a unique number.

## 4. Experimental Results

All images were preprocessed to obtain only the region of interest containing the image of the heart and resized to 224 × 224 for input to the RNet50. For each image, a feature of size 2048 was generated (i.e., *LDPRes*). The proposed algorithm was executed using a system with the following specifications: Intel *i7* 7700 k quad-core processor @ 4.7 GHz, 8 GB 2400 MHz single-channel memory with NVIDIA GTX 1070 GPU 8 GB VRAM, under the MATLAB platform. Obtained features with *p*-values < 0.005 were selected and then ranked in descending order of *t*-values. The selected ranked features are shown in Table 1.

**Figure 8:**
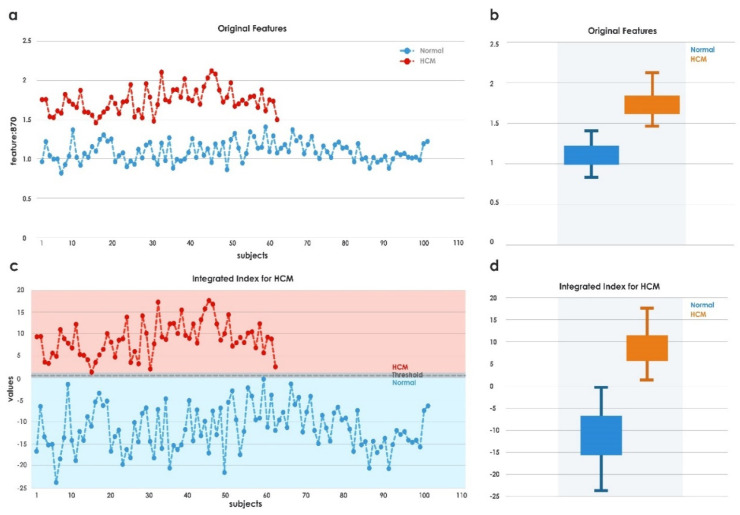
Feature distribution before and after indexing.

The highest-ranked feature, *LDPRes870,* was highly significant, and its distribution is shown in Figure 8a,b. It can be seen that both normal and HCM data were distributed toward the positive side. Hence, highly significant features of *LDPRes870* were used in Equation (5) for *IIHCM*. It was noted from Figure 8c that HCM and normal data were distributed toward the positive and negative sides, respectively. From Equation (6), the threshold value was calculated as *Th* ≈ 0.5, a single distinct number that separated the HCM versus normal cases as shown in the box plots of indexed data in Figure 8d. An integrated index above a threshold of 0.5 separated HCM from healthy subjects with 100% accuracy in our test dataset.

### Comparative Study

We compared the proposed method to four deep-learning techniques: ResNet-18 (RNet18) [29], AlexNet (ANet) [60], DarkNet (DNet) [61], and GoogLeNet (GNet) [62]. In the experiments, pretrained networks were used, as the small size of the dataset made it difficult to fine-tune the parameters. Feature extraction was performed by activating layers of the pretrained network as features. The details of each network with its generated feature size for *LDP* images are given in Table 2.

Every preprocessed image was applied with the *LDP* to obtain the local texture feature and then input to the aforementioned deep-learning architecture. Image feature sizes of 2048, 512, 4096, 1000, and 1024 were obtained using RNet50, RNet18, ANet, DNet, and GNet, respectively. These features were further ranked using Student’s *t*-test. Ranked features obtained from various methods were classified using the SVM classifier with a polynomial kernel, and various performance measures such as accuracy (Acc.), sensitivity (Sen.), specificity (Spe.), positive predictive value (PPV) were computed [63]. It was observed that *LDP*-RNet50 achieved a remarkable performance using only *three* features. Table 3 shows the performance of various methods using *three* features for a randomly partitioned training (70%) and test (30%) dataset. Though *LDP*-RNet50 with SVM achieved 100% accuracy, on the other hand, the proposed *IIHCM* categorized the HCM and normal using single integer values with appropriate positive and negative ranges.

We proposed to use *LDP* derived from US images for training in preference over raw US images because we believed *LDP* would be more stable in the presence of noise and changes in image brightness, which are common quality issues with US. As shown above, the *LDP*-RNet50 combination achieved the best performance in our experiments. To assess the contribution of *LDP*, we conducted additional experiments using RNet50, as well as various customized CNNs—CNN-1 and CNN-2, with 12 and 16 layers, respectively [18]. With each learning method, the US image dataset was randomly partitioned into 70% training and 30% testing data, and the system was tested 10 times. The average accuracy rates were 89.65%, 92.51%, and 93.26% for CNN-1, CNN-2, and RNet50, respectively, which were good, but still lower than the *LDP*-RNet50 approach.

## 5. Discussion

**Figure 9:**
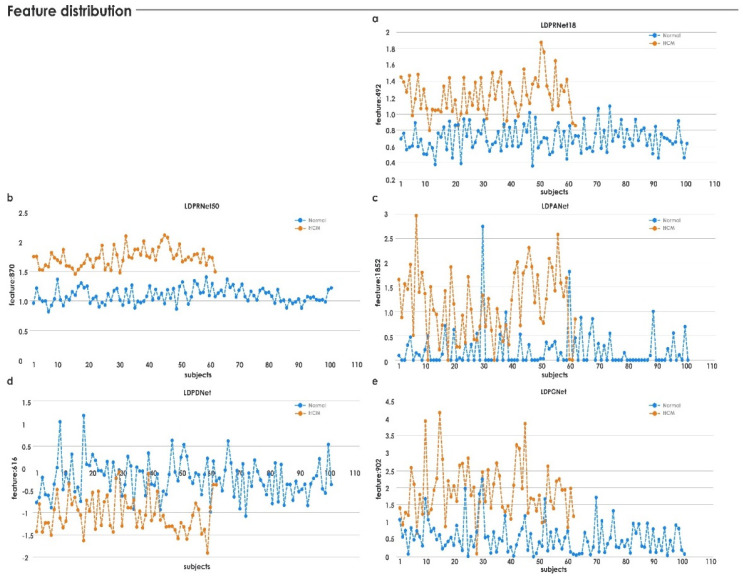
Distributions of the highest-ranked features obtained from the various methods.

In this study, the categorization of normal versus HCM was conducted on 163 heart US images. US image quality is often degraded by noise, and image brightness may affect interpretation, which scan settings may arbitrarily alter. We used *LDP* to obtain reproducible structural patterns. It encoded the texture by employing different directional responses. In the presence of noise, the relative perspective of edges may change. In such situations, *LDP* produces more stable patterns. *LDP*-based images were input to the RNet50 network to obtain deep features. This process yielded good separation results (see Figure 8). The use of a pretrained model allowed us to extract the features from our dataset with a fixed mechanism using pretrained weights [40], which was faster than training the model with random weights [64]. The generated deep features were clinically significant, with *p*-values < 0.005. The highest-ranked features from all methods were considered, and their distributions are shown in Table 4 and Figure 9, respectively. In contrast to the *LDP*-RNet50 feature, which exhibited good separation, all features from the other deep-learning approaches demonstrated overlapping between the HCM and normal groups (see Figure 9).

**Figure 10:**
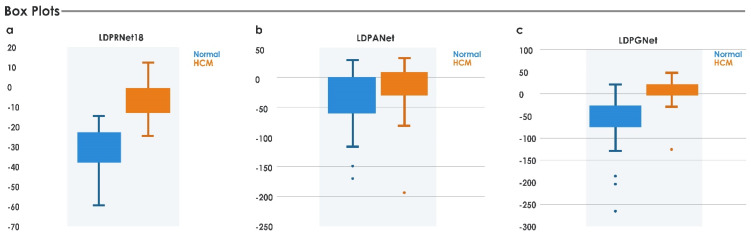
Index application to RNet18, ANet, and GNet features.

Using Equations (5) and (6), a single number was formulated for each feature that could distinguish HCM from normal subjects. *IIHCM* was also applied to other deep features (Table 4), but the results from RNet18, ANet, and GNet did not surpass those derived from the RNet50 network (Figure 10), and could not discriminate as well between healthy versus HCM subjects. The box plot for DNet is not shown due to its negative values.

**Figure 11:**
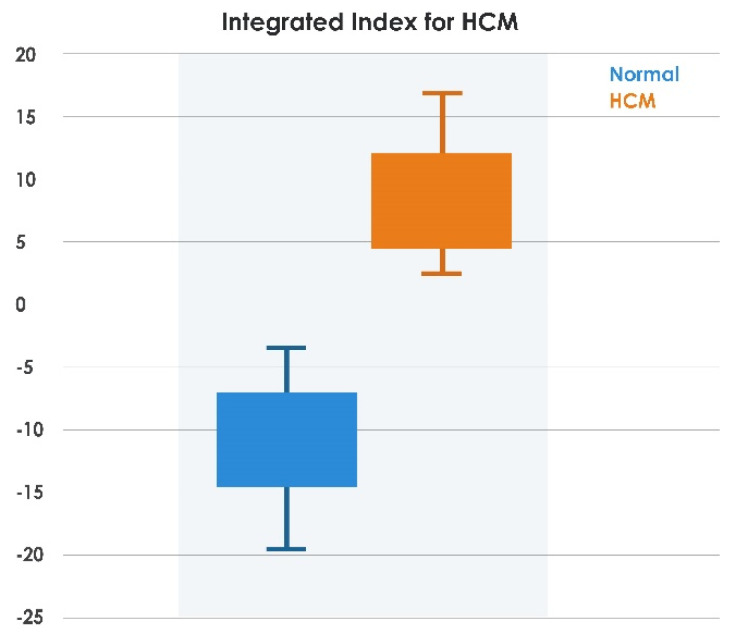
Classification using proposed *IIHCM* methodology with the *LDPRes870* feature on divided training (70%) and testing data (30%).

Our best results and integrated index were obtained by training the entire *LDP* dataset using the pretrained RNet50 model without fine-tuning. Then, as a sensitivity analysis, we divided the *LDP* dataset into 70% training and 30% testing sets (which would have been a more conventional approach had the learning method not been pretrained), and repeated the experiment. The integrated index thus derived using identical methodology demonstrated excellent separation between normal and HCM (Figure 11), which indicated that our findings were robust.

**Figure 12:**
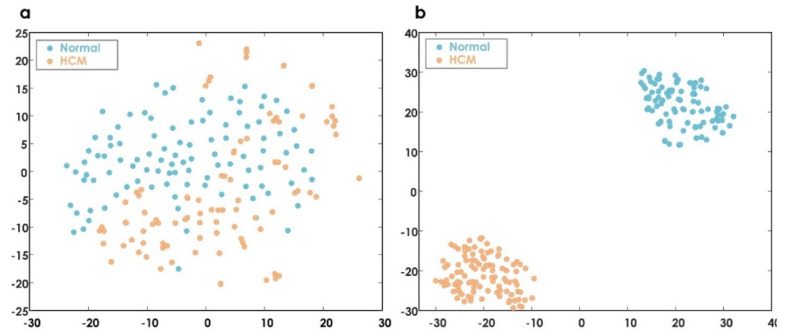
(**a**) Using complete feature space, and (**b**) using the 10 most significant *LDP*-RNet50 features.

We synthetically generated minority class samples using an adaptive synthetic (ADASYN) sampling approach [65]. ADASYN uses the weighted distribution of minority data and reduces the bias that is introduced due to an imbalanced dataset. As a result, we obtained 198 samples (i.e., normal = 101 samples and HCM = 97 samples) after ADASYN. Further, the obtained *LDP*-RNet50 features of all the samples were analyzed using the t-distributed stochastic neighbor embedding (t-SNE) technique, which helped to visualize data by reducing its dimensions [66]. Figure 12 shows the data visualization of ranked *LDP*-RNet50 features using t-SNE.

**Figure 13:**
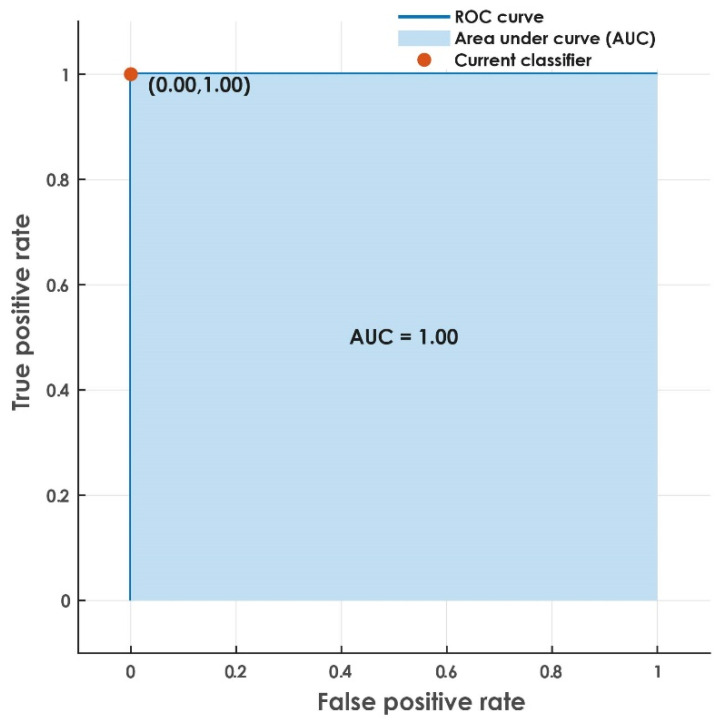
Receiver operating characteristic (ROC) curve obtained for the proposed approach.

Further, we performed classification using the *k*-fold cross-validation technique. The SVM classifier obtained an accuracy of 100%, a sensitivity of 100%, and a specificity of 100% with 10-fold cross-validation (the obtained results were identical when *k* = 5 and 7). In addition, it was noted that the proposed system achieved an area under the curve (AUC) of 1.00 (Figure 13).

In addition, we used various criteria such as entropy, Bhattacharyya, ROC, and Wilcoxon to access the significance of the generated features [67,68]. It was observed that only Student’s *t*-test and the Wilcoxon signed-rank test determined feature *LDPRes870* to be the most significant feature, with *p* < 0.005 (refer to Table 4 and Table 5).

However, using these features, the proposed *IIHCM* achieved an accuracy of 100%. Table 6 summarizes the state-of-the-art techniques proposed to detect HCM using four-chamber heart US images. To the best of our knowledge, this is the first work to propose the index for HCM classification.

This work successfully categorized normal versus HCM heart US images. The advantages of the proposed system are:

- An integrated index based on heart US image features was developed that could effectively discriminate for HCM subjects.
- The use of a single distinct value simplified the classification and should garner early clinical adoption, especially in rural and semiurban areas where access to experienced US operators may be limited.
- The proposed framework can be generalized to image analysis of other imaging modalities and/or other anatomical regions; e.g., fundus images, brain magnetic resonance imaging, etc.

We have shown that the novel combination of *LDP* and RNet50 helped to extract the discriminative features automatically without the need for manual input; e.g., measurement of dimensions or quantitation of degree of curvature. The excellent performance appeared to be unique to the combination. It was observed that *LDP* combined with other learning methods such as ResNet-18, AlexNet, DarkNet, and GoogLeNet did not yield a good separation of the features. Learning of original US images with RNet50 and without *LDP* processing yielded inferior performance. Our novel *LDP*-RNet50-based method represents an original contribution toward the automatic classification of HCM versus normal from images, instead of traditional methods requiring at least some expert knowledge and input. In addition, we have developed from the *LDP*-RNet50 an integrated index that can distinguish HCM from normal based on a diagnostic threshold value that we derived from our dataset. Unlike simple binary classification, an index is a relatable parameter that can inform the doctor of how close to the classification threshold value an individual patient’s analyzed US image would be, which may influence clinical decisions for repeat confirmatory assessment, especially in cases with borderline index values. An index with a threshold value thus carries intuitive appeal for the clinician, and features-derived indices in diverse applications have been reported in the literature [50,51,52,53,54,55,56,57,58,59]. This model was developed using 163 images. It is a prototype developed using images taken from one center (i.e., Kasturba Hospital Manipal, Manipal). Before deploying for clinical use, the developed model needs to be validated with more images collected from other centers, which is a topic for future work. Herein, we have focused on developing a novel index to discriminate HCM patients from normal patients. HCM is generally depicted as a distinct cardiomyopathy. Numerous pathologies can cause left ventricular hypertrophy, such as hypertension, chronic kidney disease, athlete’s heart, etc. The gold standard diagnostic test is still genetic testing. In this work, automated detection of HCM was a revolutionary approach in the noninvasive diagnosis of this rare disease.

The limitations of the proposed system are:

The proposed work categorized sample data into HCM or normal with high accuracy, but did not consider the discernment between HCM and other causes of hypertrophy, which is clinically relevant. It also did not classify the images according to the grade of hypertrophy severity. The method should be independently validated, preferably with larger datasets from multiple centers, before it can be clinically adopted. We plan to address the possible uncertainty issue in our developed model by acquiring more images from various centers in our future studies. The system did not classify the types and extent of hypertrophy patterns among HCM patients. In addition, functional assessment and their role in prediction of complications associated with the HCM were not studied.

## 6. Conclusions

Hypertrophic cardiomyopathy is a genetic disease of the heart. The generated *IIHCM* helps to identify the HCM cases with a single threshold value. The proposed indexing entails easy classification that dispenses with the need to manually label the images. The results of the current study are promising and can stimulate new studies using different techniques and more extensive datasets. This approach can help to identify the disease, but when employed for serial monitoring, it can assist in understanding disease longitudinal progression. The limitation of this work is that it was developed using a small dataset. We plan to validate our work with images collected from different centers in the future. We plan to extend the work with more heart US images to characterize various diseases, including ischemic heart disease and other causes of the hypertrophied left ventricle, such as hypertensive heart disease, etc.

## Figures and Tables

**Table 1:** Ranked Features with Means, Standard Deviations (SD), and *p*- and *t*-Values.

Features	Normal		HCM		*p*-Value	*t*-Value
	Mean	SD	Mean	SD		
*LDPRes870*	1.0906	0.1285	1.7401	0.1585	5.1 × 10^−65^	28.6105
*LDPRes1731*	1.6194	0.3706	2.7880	0.3472	1.58 × 10^−45^	20.0121
*LDPRes1313*	2.121	0.3118	1.3068	0.2749	1.57 × 10^−37^	16.9102
*LDPRes1701*	0.8078	0.1347	0.4998	0.0998	6.62 × 10^−34^	15.5594
*LDPRes1100*	0.9853	0.1107	1.2622	0.1160	5.59 × 10^−33^	15.2183
*LDPRes54*	0.0675	0.0336	0.1584	0.0438	4.1 × 10^−32^	14.9010
*LDPRes110*	1.1768	0.2628	1.8776	0.3561	9.59 × 10^−31^	14.4011
*LDPRes1351*	0.7101	0.2133	0.3076	0.1133	7.92 × 10^−29^	13.7051
*LDPRes223*	0.1786	0.0606	0.0634	0.0351	1.58 × 10^−28^	13.5969
*LDPRes770*	2.4266	0.2920	1.8258	0.2951	4.83 × 10^−26^	12.6988

**Table 2:** Architecture details of the various deep-learning methods used in this work.

Parameters	RNet50	RNet18	ANet	DNet	GNet
Input image size	224 × 224 × 3	224 × 224 × 3	227 × 227 × 3	256 × 256 × 3	224 × 224 × 3
No. of deep layers	50	18	8	19	22
Output layer	‘avg pool’	‘pool5′	‘pool5′	‘avg1′	‘pool5-7 × 7′
No. of features	1 × 2048	1 × 512	1 × 4096	1 × 1000	1 × 1024

**Table 3:** Performance of various methods.

Methods	Acc. (%)	Sen. (%)	Spe. (%)	PPV (%)	F-Score
*LDP*-RNet18	95.12	98.38	94.05	91.04	0.9456
*LDP*-RNet50	100	100	100	100	1
*LDP*-ANet	87.11	82.25	90.09	83.60	0.8291
*LDP*-DNet	84.04	83.87	84.15	76.47	0.7999
*LDP*-GNet	93.25	90.32	95.04	91.80	0.9105

**Table 4:** Highest-ranked features using the various deep-learning approaches.

Features	Normal		HCM		*p*-Value	*t*-Value
	Mean	SD	Mean	SD		
*LDP*-RNet18						
*LDPRes492*	0.696662	0.155103	1.216885	0.231307	2.98 × 10^−38^	17.18295
*LDP*-RNet50						
*LDPRes870*	1.0906	0.1285	1.7401	0.1585	5.1 × 10^−65^	28.6105
*LDP*-ANet						
*LDPAlex1852*	0.194477	0.393206	1.13497	0.689104	1.31 × 10^−21^	11.09695
*LDP*-DNet						
*LDPDark616*	−0.21708	0.421096	−1.02086	0.402245	3.41 × 10^−24^	12.03204
*LDP*-GNet						
*LDPGoogLe902*	0.547527	0.457099	1.890254	0.790567	6.12 × 10^−29^	13.74575

**Table 5:** Highest-ranked features using various ranking methods.

Features Using Various Methods	Normal		HCM		
	Mean	SD	Mean	SD	*p*-Value
Entropy					
*LDPRes17*	0.000195	0.001399	0	0	0.274759
Bhattacharyya					
*LDPRes17*	0.000195	0.001399	0	0	0.274759
ROC					
*LDPRes28*	0	0	0.000952	0.004895	0.052053
Wilcoxon					
*LDPRes870*	1.090677	0.128562	1.740115	0.158587	5.1 × 10^−65^

**Table 6:** Summary of state-of-the-art work using echocardiogram images/videos.

Paper	Method	Result	Dataset
[19]	PCA + BPNN	Accuracy = 92.04% (normal and abnormal (DCM and HCM))	Echocardiogram videos: 60
[20]	DPSO-FCM + GLCM and DCT + SVM	For segmentation accuracy: 95% For classification accuracy: 90%	Echocardiogram videos: DCM: 40, HCM: 40, normal: 10
[21]	Multilayer CNN	C statistics: 0.93 (for HCM)	HCM: 495 studies to train the model
[22]	First-order statistics + GLCM + SVM	Studied possible texture myocardial features with *p*-value < 0.05	Transthoracic echocardiography images: HCM, uremic cardiomyopathy, and hypertensive heart disease (50 cases for each group)
**Ours**	***LDP* + ResNet-50 + ADASYN + *IIHCM***	**Accuracy: 100%**	**Echocardiography images** **Normal: 101** **HCM: 97**

## Data Availability

Not applicable.
